# Extracting sub-cycle electronic and nuclear dynamics from high harmonic spectra

**DOI:** 10.1038/s41598-021-82232-1

**Published:** 2021-01-28

**Authors:** Dane R. Austin, Allan S. Johnson, Felicity McGrath, David Wood, Lukas Miseikis, Thomas Siegel, Peter Hawkins, Alex Harvey, Zdeněk Mašín, Serguei Patchkovskii, Morgane Vacher, João Pedro Malhado, Misha Y. Ivanov, Olga Smirnova, Jon P. Marangos

**Affiliations:** 1 Blackett Laboratory, Imperial College London, Prince Consort Road, London, SW7 2AZ UK; 2 ICFO - The Institute of Photonic Science, Castelldefels, Barcelona Spain; 3 Max-Born Institute for Nonlinear Optics and Short Pulse Spectroscopy, Berlin, Germany; 4 Chemistry Department, Imperial College London, Prince Consort Road, London, SW7 2AZ UK; 5 CEISAM, UMR 6230, Université de Nantes/CNRS, 44000 Nantes, France; 6 Present Address: Institute of Theoretical Physics, Charles University, Praha 8, Czech Republic

**Keywords:** High-harmonic generation, Ultrafast photonics, Attosecond science

## Abstract

We present a new methodology for measuring few-femtosecond electronic and nuclear dynamics in both atoms and polyatomic molecules using multidimensional high harmonic generation (HHG) spectroscopy measurements, in which the spectra are recorded as a function of the laser intensity to form a two-dimensional data set. The method is applied to xenon atoms and to benzene molecules, the latter exhibiting significant fast nuclear dynamics following ionization. We uncover the signature of the sub-cycle evolution of the returning electron flux in strong-field ionized xenon atoms, implicit in the strong field approximation but not previously observed directly. We furthermore extract the nuclear autocorrelation function in strong field ionized benzene cations, which is determined to have a decay of $\tau _0 = 4 \pm 1$ fs, in good agreement with the $\tau _0 = 3.5$ fs obtained from direct dynamics variational multi-configuration Gaussian calculations. Our method requires minimal assumptions about the system, and is applicable even to un-aligned polyatomic molecules.

## Introduction

The ionization of a molecule by a strong laser field or a short x-ray pulse triggers sub-femtosecond to few-femtosecond electronic and vibronic motions^1–3^ that are at the frontier of our understanding of photo-induced chemical dynamics^4–9^. In the case of a sudden single, multiphoton, or strong field ionization qualitatively new features are expected beyond those encountered for ionization with narrow band or weak fields. The formation of coherent superpositions of electronic states becomes possible due to the large bandwidth/fast timescales of the ionization event, so that the evolution is now of *coupled* nuclear and electronic wavepackets, while the presence of the laser in strong field ionization further modifies the dynamics. Resolving these electron-nuclear dynamics with sub-femtosecond temporal and angstrom-scale spatial resolution can illuminate the role of coherent multi-electron motion in chemistry^10,11^ and the feasibility of controlling chemical dynamics through the selective excitation of electronic states^12^, and is therefore a key challenge in attosecond science.

One particularly elegant method for measuring the early time dynamics leverages the fact that when the ionized electron is removed by a strong field, light-induced charge dynamics lead to a highly nonlinear optical response – the generation of high harmonics of the incident field. Reconstructing the dynamics by analysing the emitted harmonic light is the core of high harmonic generation spectroscopy (HHGS)^4–7,13–19^, which probes the evolution of the non-equilibrium cation state after ionization via radiative recombination of the liberated electron with the hole left behind. The ultrafast evolution of the hole and the nuclear wavefunction are recorded in the radiative recombination amplitudes and mapped onto the properties of the emitted light. HHGS delivers sub-femtosecond temporal resolution due to the precise mapping between the energy of the emitted photon and the time delay between ionization and recombination^20,21^. The harmonic emission is determined by the ionisation dipole, the recombining photoelectron wavepacket, the recombination dipole, the relative amplitudes and phases of all participating channels, and the nuclear evolution that damps the emission with increasing return time^22^.

Separating the many contributing factors requires multi-dimensional techniques, whereby harmonic spectra are recorded whilst field or molecular parameters are varied; these approaches have had success with simple systems such as H$_2$, CH_4_, CO_2_ and N_2_. Molecular alignment relative to laser-field is especially effective^17,23^, but is strongly limited to small molecules. Isotopic substitution^4,24,25^ has proven effective for measuring the nuclear autocorrelation function (the overlap between the evolving and initially-prepared nuclear wavepacket^26^), but is limited by the availability of appropriate isotopes. For example, in the present work we found no significant difference between HHG in benzene and deuterated benzene (see SM sect. S1), indicating that isotopically substituting the hydrogen atoms is insufficient to affect the relevant nuclear dynamics in these molecules. All-optical methods are more generally applicable; for instance, adding a weak second harmonic to the driving field and scanning the relative phase between them has proved effective in extracting the relative phases and amplitudes of the quantum paths associated with different ionization-recombination channels^14,27,28^, though to date it has not been capable of extracting the nuclear autocorrelation function.

The challenges of other multidimensional HHGS techniques^29^ have motivated us to develop a simple two-dimensional technique, capable of measuring ultrafast nuclear and electronic dynamics from unaligned samples, which places no requirements on the sample. Our method is based on measuring the harmonic spectra simply as a function of the laser intensity. By decomposing the two-dimensional map of harmonic spectrum vs. intensity, we extract the returning electron wavepacket flux and nuclear autocorrelation function, all from unaligned samples. We demonstrate our method by measuring, for the first time, the returning electron flux in strong-field ionized xenon atoms and the nuclear autocorrelation time in benzene, obtaining good agreement with state-of-the-art calculations.

## Results

**Figure 1:**
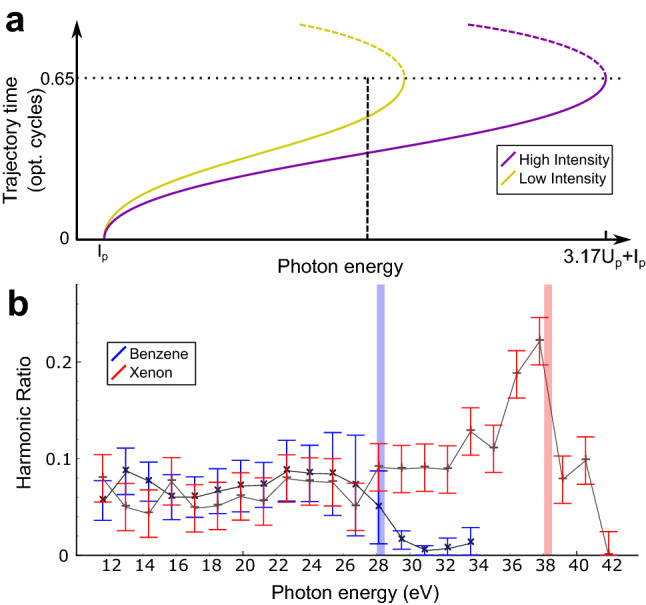
(**a**) Mapping of emitted photon energy to trajectory time for two different laser intensities, purple (higher) and yellow (lower). The “short” photoelectron trajectories recombining within 0.65 of the laser-cycle duration (solid lines) will contribute to the on-axis harmonic emission. Trajectories recombining at later times (dashed lines) do not contribute. The vertical dashed line shows two different evolution times being sampled at a single photon energy. (**b**) Low-to-high laser intensity ratios of harmonic yields in benzene (blue, 15 TW/$\hbox {cm}^2$and 35 TW/$\hbox {cm}^2$) and in xenon (red, 25 TW/$\hbox {cm}^2$and 40 TW/$\hbox {cm}^2$). The vertical shaded regions indicate the classical cutoffs corresponding to the lower intensities. Error bars reflect plus and minus one standard deviation of the ratio across six repetitions in benzene, restricted to positive values.

Our method is based on the dependence of the intrinsic time-energy mapping in HHG upon the driving laser intensity. The time-delay between ionization and recombination in HHG is fixed between 0 to 0.65 laser cycles assuming short-trajectory phasematching, with the harmonic emission energies ranging from $I_{\mathrm{p}}$ and $1.3I_{\mathrm{p}}+3.17U_{\mathrm{p}}$, where $U_{\mathrm{p}}=I_L/(4\omega _L^2)$ is the average quiver energy of the electron in the laser field for a field with intensity $I_L$ and frequency $\omega _L$. Expressions are in atomic units unless noted. Increasing the intensity causes the same time range to be mapped to a larger range of photon energies as shown in Fig. 1a, effectively separating ‘static’ factors, defined here as those which depend on the harmonic frequency only, from the ‘dynamic’ factors which depend on the laser intensity and the timing of the ionization and recombination events. Simply taking the ratio of HHG spectra from the same species at different intensities cancels out static factors (namely the dipole recombination factor), giving access to the dynamical information. For a given harmonic order increasing the intensity decreases the corresponding time-delay between ionization and recombination; comparing the trend across all harmonics as the intensity is increased then allows us to extract a self-consistent time behaviour for the cation.

In particular, the ratio of harmonics produced at low and high laser intensities, henceforth denoted *low-to-high* ratio, is the ratio of the dynamic portion of the signal to a stretched-in-time version of itself since the continuum motion is *self-similar*, so the varying intensity simply means the temporal-window is mapped to a wider range of photon energies. Additionally, changing the laser intensity changes the multi-electron dynamics in the cation, but this change can be accurately incorporated in the analysis^14^. Because the dynamic factors are typically smooth, the dynamic factor at a sufficiently high laser intensity is approximately constant across the spectral range taken at lower laser intensity, and the low-to-high ratio qualitatively reflects the dynamics.

Figure 1b presents low-to-high ratios measured in benzene and xenon acquired with an 1800 nm laser, carefully arranged to satisfy the short-trajectory phasematching conditions (see Methods). The low-to-high ratio in xenon extends to higher photon energies because the ionization potential of xenon (12 eV) is higher than that of benzene (9 eV), meaning slightly higher laser intensities are needed in xenon to get comparable signal levels, leading to different cutoff energies. In xenon, the ratio increases with frequency with increasing slope, rising to a pronounced peak at the classical cutoff (38 eV). As discussed below, this is exactly the prediction from the standard strong field picture of HHG, and the effect should be present in the HHG emission of all atoms and molecules^30^. By contrast, in benzene the low-to-high ratio at the cutoff (28 eV) is smaller and smoother, never developing the peak structure. This observation indicates that in benzene there is an additional dynamical factor from the nuclear or electronic evolution in the cation causing significant attenuation in the vicinity of the cutoff, near delays of 0.65 optical cycles.

This qualitative picture can be made quantitative through a physically motivated analysis of the 2D spectra by fitting the yield to the three-factor ansatz

1 $$\begin{aligned} S(\omega ,I_{\mathrm{L}})=A(\omega ) B(I_{\mathrm{L}}) C(\bar{\omega }). \end{aligned}$$

The static factor $A(\omega )$ depends on the harmonic frequency $\omega$, and encodes the photorecombination dipole averaged over participating orbitals and molecule angles, as well as any uncompensated spectral response of the experimental apparatus. The ionization rate factor $B(I_{\mathrm{L}})$ depends on the laser intensity $I_{\mathrm{L}}$ and encodes the angularly and orbital-averaged tunnel ionization rates. The dynamic factor $C(\bar{\omega })$ depends on $\bar{\omega }=\frac{\omega - 1.3I_{\mathrm{p}}}{U_{\mathrm{p}}}$, the emission frequency normalized to the classical cutoff so that the cutoff occurs at $\bar{\omega }\approx 3.17$. This normalized frequency has a one-to-one mapping to the trajectory time for a given trajectory type (either short or long)^21,26,27^. This empirical factorization (1) is similar to “quantitative rescattering theory” (QRS)^31^ in that it involves separating a frequency-dependent recombination factor, but unlike in QRS we also separate the role of cycle-averaged tunnel ionization ($B(I_{\mathrm{L}})$) from sub-cycle factors ($C(\bar{\omega })$).

**Figure 2:**
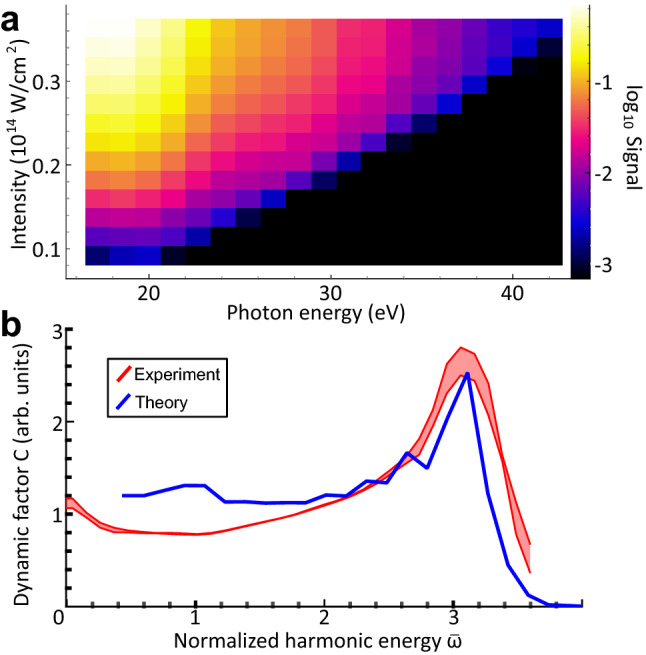
(**a**) An example of a two-dimensional intensity versus HHG spectrum recorded for benzene. Note the linear scaling of the cut-off with intensity in this non-saturated measurement regime. (**b**) Dynamical factor *C* obtained from fitting to experimental (red) and simulated (blue) HHG intensity scans in xenon. The experimental shaded area for xenon represents the mean plus/minus one standard deviation of the fits obtained by varying the numerical parameter $N_C$ (see SI sect. S2).

Our fitting technique reduces all dynamic processes, such as nuclear motion and the interference of multiple electronic states, into a single observable function $C(\bar{\omega })$ comparable to theoretical calculations. To apply the method we obtain two-dimensional data sets comprising the HHG spectrum as a function of laser intensity for xenon and benzene; see Fig. 2a for an example. The laser intensity was varied controllably using a pre-calibrated adjustable iris in the laser beam, providing robust and reproducible control of the relative intensity. Determining the absolute peak intensity in strong-field experiments to high precision is known to be very challenging; we thus repeated the fitting procedure for a range of assumed peak intensities and the correct value for the absolute intensity can then be inferred by agreement with theoretical calculations. Such a global fitting procedure could be avoided by more precise absolute peak intensity determination in future work. Further details of the fitting procedure and the quality of the fit are given in Supplementary Information section S2. Figure 2b shows the results of one such fit, the experimental dynamic factors for xenon (red). The shape of the curve, with pronounced peak at the cutoff, is qualitatively consistent with the low-to-high ratios (Fig. 1b), but we can now quantify the height of the cutoff peaks.

We use a detailed numerical model, namely extended strong-field approximation (SFA)^5,13,32–34^ plus macroscopic averaging, to calculate theoretical two-dimensional spectra, to which we can also fit equation 1 (see Methods). The theoretical result for xenon is plotted in Fig. 2b in blue. There is good agreement between the xenon experimental and theoretical curves, showing we accurately capture the various factors that contribute to *C* in an atom, where nuclear dynamics are absent. The peak near the cutoff can be explained through the sub-cycle behaviour of the electron current as described by the three-step model – the cutoff is the peak of the velocity distribution of the recolliding electrons^30^. This effect is compounded by the higher laser field amplitude at birth times corresponding to the cutoff compared to the plateau, which results in a higher tunnel ionization rate^35^. In this way we extract the sub-cycle evolution of the recolliding electron flux, implicit in the strong field approximation but not previously observed directly.

**Figure 3:**
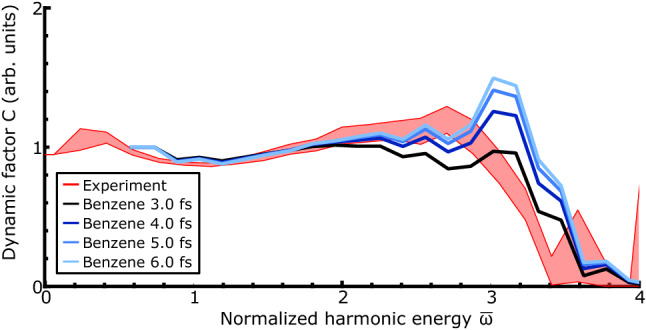
Dynamical factor *C* obtained from fitting to experimental (red) and simulated (blue-black) HHG intensity scans for benzene. The experimental shaded region for benzene represents 10–90% percentile intervals from bootstrapping the fit procedure using 6 independently recorded datasets and the simulated curves correspond to different decay constants $\tau$ as shown in the legend.

The measurement of the returning electron flux is a benchmark result; because of the near universality of the returning electronic wavepacket, deviations from this shape can be primarily attributed to the internal dynamics of a system under study. Applying the method to a system with nuclear dynamics radically modifies the results in a transparent way, as exhibited by the result obtained from benzene in Fig. 3 where the cutoff peak is strongly suppressed. Benzene is an ideal model case as its high symmetry precludes good molecular alignment and, as previously stated, no significant differences are observed upon deuteration. Nevertheless, our method will allow us to extract the nuclear autocorrelation. The degeneracy of the HOMO and HOMO-1 together with the order of magnitude change in tunnelling rate between HOMO and HOMO-2 leads to dynamics well described by a single electronic state. The minimal contribution of lower lying states can also be seen in the lack of higher energy secondary cutoffs. The striking difference between the xenon and benzene experimental C curves suggests that dynamic attenuation of HHG by factor of $\approx 2$ occurs in the first 4 fs (the maximum short trajectory duration) after ionization in benzene compared to xenon. Though different orbital structure and ionization energies of the two species mean that simulation is necessary to quantitatively attribute this attenuation to cation evolution, there is already a distinct qualitative difference. To quantitatively extract the contribution of cation evolution in *C*, we simulate the experiment with different assumed nuclear autocorrelation decay times and fit eq. 1 to the results. Therefore for a molecule, we extract dynamics by comparing the experimental dynamical factor *C* with that of a suitably parametrized model, in this case the decay time of the nuclear autocorrelation. As the factor *C* contains all dynamical information for other systems parametrization in terms of electronic and nuclear dynamics may be more appropriate, but our simulations show that in benzene the nuclear dynamics dominate. Recalling that we perform an optimization of peak intensity for the experimental data, this means we search for a minimum in the difference between theory and experiment as a function of these two parameters. Here we present data varying the autocorrelation time for the optimal peak intensity. Setting $\tau _0=\infty$ the dynamic factor shows a peak around the cutoff which is inconsistent with the experiment. Introducing an autocorrelation function with decay time of 4 fs reduces this peak and makes the simulated dynamic factor have a similar shape to the experimental curve. Further reduction to 2 fs causes the dynamic factor to drop monotonically, inconsistent with the experimental curve. Thus we can determine the magnitude of the nuclear autocorrelation function in benzene to be $\tau _0 = 4 \pm 1$ fs, with the uncertainty originating from confidence intervals on the experimental *C* which are determined by bootstrapping with six different datasets. This method retrieves the nuclear autocorrelation function in the presence of the strong field, with an unaligned sample, without recourse to isotopic substitution, for the first time.

## Discussion

Our analysis of the experimental data places the nuclear autocorrelation decay time around 4 fs (with uncertainties around $\pm 1$ fs). We compare this result to simulations of the nuclear dynamics using the direct dynamics variational multi-configuration Gaussian method^36–38^ (see Methods). The simulation predicts a decay time of 3.5 fs which compares well to the measured value, even though the theory does not include the strong field. This good agreement allows us to describe the few-femtosecond nuclear motion in detail through the model. Initially, the neutral benzene molecule is planar and symmetric, with the six C–C bonds 1.40 Å long. Upon ionisation to the lowest cationic state, the molecule stays planar but the bond lengths evolve with time. Specifically, two opposite C–C bonds shorten by approximately 0.02 Å in 5 fs. The other four C–C bonds lengthen by approximately 0.02 Å over the same time period. These dynamics lead the molecular cation to acquire a more stable quinoid structure in equilibrium with the new electronic configuration^39^. All the C–H bonds shorten slightly but the absolute magnitude of the variation is almost 3 times less than for the C–C bonds. Because the lowest-energy states of benzene cation result from ionisation of the carbon $\pi$ system, much lower distortion of the C–H bonds is expected. This behaviour is consistent with our experimental observation that no significant differences were found in the present work between benzene and deuterated benzene. We note that our work shows the harmonic spectra is strongly modulated by C–C bond motion, as opposed to significantly faster hydrogen motion as in previous studies, pointing towards the ability of HHGS to shed light upon important electronic-nuclear couplings at few-femtosecond timescales.

While here we have focused upon the nuclear dynamics in benzene cations, where there is anticipated to be a single dominant cation channel in HHG, we are not limited to this case. A generalisation of the method to cases where multiple cation states play a role is possible by modifying equation 1 to a sum over these states. This generalization does present some challenges; the experimental data can no longer be directly factorized, and instead we need to find a series of such factorizations. This problem is formally identical to decomposing the data, a high order tensor, into a sum of rank 1 tensors (a rank decomposition). While a formal solution to this problem is not known at present, we note that tensor decomposition is a widely used tool in machine learning, where it is used to find the underlying physics behind a data set^40^. Our situation, in which the underlying physics is known and we seek the exact decomposition, is significantly simpler and so this approach seems promising. An alternative approach to direct decomposition, but still in the spirit of our factorization, would instead be to directly compare the experimental data to a 2D calculation. Calculating and minimizing an error score then forms the crux of the method, and by making the error metric independent of the intensity-dependence of the total yield and spectrum we operate in the spirit of the method described above, in particular removing any dependence on a realistic ionization rate or any experimental spectral sensitivity. The error therefore represents the discrepancy in the non-factorable part of the intensity-dependent spectrum, which contains dynamical information. This approach suffers from requiring a more complete theoretical model, but is more general.

Both approaches place more stringent requirements on the signal-to-noise than the direct factorization. Improved experimental data are expected using recently developed $\approx$100 kHz repetition rate MIR OPCPAs, improving acquisition times by a factor of 100 compared to the data here. Our method, and the extensions to multiple cation states, are furthermore straightforwardly combined with other multidimensional spectroscopies; for instance one could combine intensity scanning with $\omega _L$/2$\omega _L$ phase scans to fully retrieve the cation dynamical effects and other electronic dynamical effects associated with excitation of multiple cationic channels. Equally a variant of the method could be used where a varying laser wavelength is used to obtain the multi-dimensional data in which the dynamics are encoded, with either fixed or scanned intensity.

In conclusion, we have developed a robust strategy for extracting dynamics from HHG spectra of polyatomic molecules recorded at a range of laser intensities. The method is experimentally straightforward and most importantly applicable to any molecule that can be delivered into the gas phase at sufficient density, without need for alignment or isotopic substitution. From the two-dimensional data we extract a function that encodes the dynamical information in an intuitive way that can be compared directly with theory. Using this method we measure the returning electron flux in HHG for the first time, and determine quantitatively the nuclear autocorrelation function in benzene cations under strong field exposure. Excellent agreement with recent theoretical predictions enables us to describe quantitatively the first few femtoseconds of nuclear evolution in benzene. This method is general and should be extendible to any polyatomic molecule, with or without molecular alignment and multiple cationic states, and greatly increases the range of molecules to which HHG spectroscopy can be applied.

## Methods

### Experimental spectra

Measurements were made using an 1800 nm laser field (the idler of an OPA system pumped by a Ti:sapphire CPA) with a pulse duration $\approx 40$ fs, spatially filtered with a hollow core fibre and focussed into a thin gas jet, with thickness ($\approx 500$ µm) much smaller than the confocal parameter. Molecular vapour continuously flowed into the vacuum; the generated HHG spectra were measured using a flat-field X-ray spectrometer. Great care was taken to ensure that spectral reshaping due to longitudinal phase mismatch or absorption was not present; these measures are further described in ref.^41^.

### Theoretical spectra

We combine an extended form^5,13,32–34^ of the strong-field approximation (SFA)^20^ for the single-molecule response with macroscopic averaging over the transverse profile of the laser beam. The SFA model is appropriately modified to handle molecular HHG as follows: we numerically integrate over return and birth time, so that tunnel ionization is captured at the SFA level of detail. Multiple cation states can be included in the calculation. A phase offset $\phi _{\mathrm{i}}$ between the emission from non-degenerate HOMO and HOMO-1 could be added. This is not included in benzene because the two states are degenerate and have very similar orbitals (reflecting the high degree of symmetry of benzene), so their contributions to the dipole are nearly identical. Adjusting the relative phase of their contributions merely changes the total intensity of the spectrum. Nuclear motion is described by a Gaussian autocorrelation function $g(\tau ;\tau _0)=\exp (-(\tau /\tau _0)^2/2)$ with decay time $\tau _0$. More complex forms of evolution can be included, but are generally not necessary for short-time scales^42^. The microscopic model is used to calculate the angle averaged local dipole response at all points throughout the interaction region, and the far-field distribution of the macroscopic response calculated by integrating across this volume. We repeat the calculation for a variety of laser intensity to form an intensity-dependent spectrum, then apply equation (1). Full details are given in supplementary information section S3.

### Nuclear autocorrelation calculations

The coupled nuclear and electron dynamics were simulated fully quantum mechanically, including all coordinates, with the “direct dynamics-variational multi-configuration Gaussian” (DD-vMCG) method^36,37^ implemented in the Quantics package^38^. This approach describes the molecular wave packet using a time-dependent basis set of variational coupled Gaussian functions. In the present simulations, a set of 12 Gaussian basis functions was necessary to obtain converged dynamics over the first 5 fs. The electronic structure was calculated on-the-fly using the multi-reference method ‘complete active space self-consistent field’ (CASSCF) and 6-31G* basis set. We chose the active set consisting of the 6 $\pi$ orbitals, sufficient to describe the lowest-energy electronic states. The quality of the potential energy surfaces had been validated against explicitly correlated MRCI method with cc-pVTZ-F12 to reach the basis set limit (see SI in ref. ^43^). Simulations include the two lowest electronic states of the cation, and are performed on a diabatic electronic basis obtained through a regularization procedure^44^ around the minimal energy conical intersection. All non-adiabatic effects between the two lowest-energy electronic states are taken into account. The initial conditions correspond to a Franck-Condon ionization of the neutral species on the ground vibrational state (in the harmonic approximation), resulting in a diabatic cationic electronic state.

## Supplementary Information

Supplementary Information.

## Data Availability

All data underlying the findings of this paper are available upon reasonable request from the corresponding authors.

## Code availability

All code underlying the findings of this paper are available upon reasonable request, licenses permitting, from the corresponding authors.
