# Supplementary material for: Extracting sub-cycle electronic and nuclear dynamics from high harmonic spectra
Source: Sci Rep. 2021 Jan 28;11:2485. doi: 10.1038/s41598-021-82232-1 (PMC7844012; doi:10.1038/s41598-021-82232-1)
Supplement: Supplementary file 1 — Supplementary Information. [file 41598_2021_82232_MOESM1_ESM.pdf]

Supplementary Information: Extracting sub-cycle electronic and nuclear dynamics from high harmonic spectra

Dane R. Austin[1], Allan S. Johnson[1,2], Felicity McGrath[1], David Wood[1], Lukas Miseikis[1], Thomas Siegel[1], Peter Hawkins[1], Alex Harvey[3], Zdeněk Mašín[3,+], Serguei Patchkovskii[3], Morgane Vacher[4,†], João Pedro Malhado[4], Misha Y. Ivanov[3], Olga Smirnova[3], and Jon P. Marangos[1,*]

[1]Blackett Laboratory, Imperial College London, Prince Consort Road, London SW7 2AZ, UK
[2]ICFO - The Institute of Photonic Science, Castelldefels (Barcelona), Spain
[3]Max-Born Institute for Nonlinear Optics and Short Pulse Spectroscopy, Berlin, Germany
[4]Chemistry Department, Imperial College London, Prince Consort Road, London SW7 2AZ, UK
[*]j.marangos@imperial.ac.uk
[+]Current address: Institute of Theoretical Physics, Charles University, Praha 8, Czech Republic
[†]Current address: Université de Nantes, CNRS, CEISAM UMR 6230, F-44000 Nantes, France

S1 Comparison of harmonic spectrum from normal and deuterated benzene

We observed no significant differences between normalized harmonic spectra from benzene and deuterated benzene. Figure S1 compares typical spectra from the two molecules with laser intensity 0.28×10^{14} W/cm^2. Any differences were at the 10% level or at the high photon energy range of the spectrum where small laser intensity fluctuations translate into large signal changes.

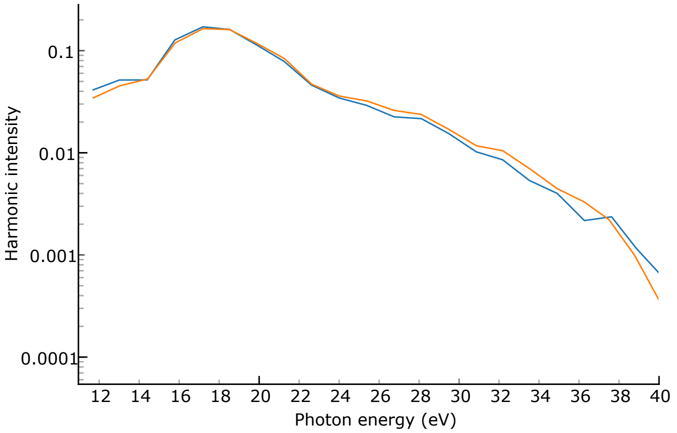

Figure S1. Measured harmonic spectrum from benzene (blue) and deuterated benzene (orange) with a laser intensity of 0.28×10^{14} W/cm^2 and wavelength 1.8 µm.

S2 Fits to harmonic spectrum laser intensity scan

The measured laser-intensity-dependent harmonic spectra are fit to eq. (1) in the main text using using an iterative procedure. Each iteration consists of minimizing the error with respect to one of the three factors while holding the other two constant. The algorithm cycles through the three factors until convergence; this typically takes less than ten minimizations with respect to each factor. Numerically, the static and intensity factors, $A(\omega)$ and $B(I_L)$ are sampled at the experimental values of ω and I_L respectively. Since $\bar{\omega}$ takes a unique value for each (ω, I_L), $C(\bar{\omega})$ must be described using a basis to control the number of degrees of freedom. We use simple linear interpolation: $C(\bar{\omega})$ is stored on a uniformly spaced vector of $\bar{\omega}$, and linear interpolation used to evaluate it at the required values of $\bar{\omega}$. The number of samples N_C is between 20–30. This procedure is

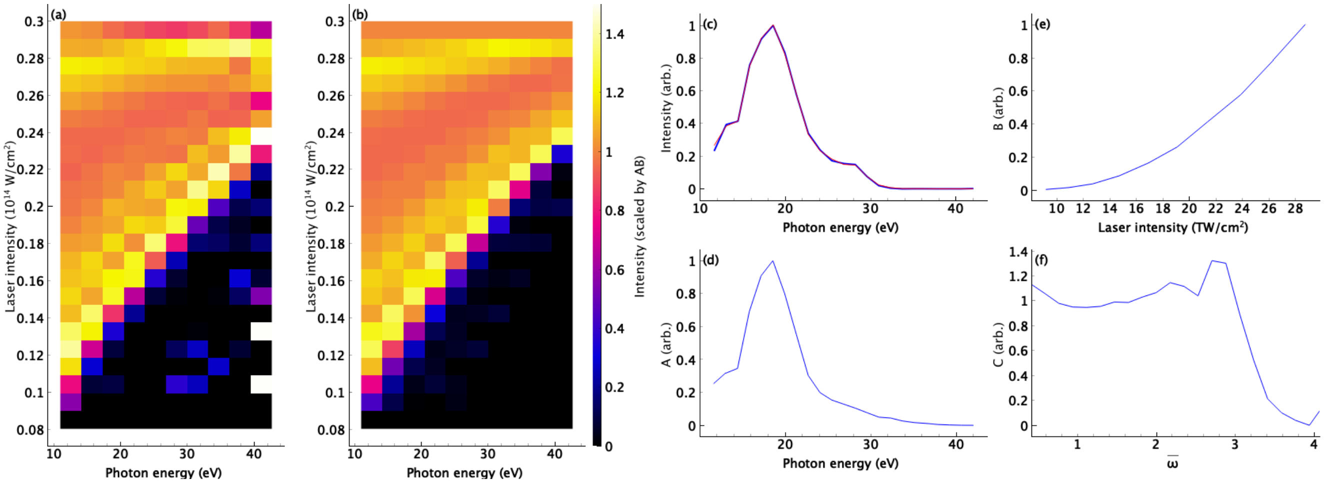

Figure S2. Measured (a) and retrieved (b) on-axis harmonic yield in benzene. The yield in both panels is scaled by the fitted static ($A(\omega)$) and intensity-dependent ($B(I_L)$) factors to improve readability. (c) Measured (blue) and fitted (red) harmonic spectra at 18 TW/cm^2. (d) Fitted static factor A. (e) Fitted ionization rate factor B. (f) Fitted dynamic factor C.

similar to finding the best rank-1 approximation to the rank-3 tensor $S(\omega, I_L, \bar{\omega})$[1,2]. We use stability of the results with respect to small changes in N_C as a measure of the quality and robustness of the fit.

The fit is of high quality for all presented intensity scans. Figure S2(a) shows a representative measured dataset and Fig. S2(b) shows its corresponding fit for comparison. All significant features are captured by the fit. For clarity, the data are scaled by the product of the static ($A(\omega)$) and intensity-dependent ($B(I_L)$) factors, which means the experimental data at high photon energy but low laser intensity appears very noisy in Fig. S2(a). For a more quantitative comparison, Fig. S2(c) compares the experimental and fitted spectra (blue and red) at 18 TW/cm^2, a mid-range intensity. The two curves are nearly indistinguishable — significant differences emerge only below 10 TW/cm^{2}due to low signal. Figure S2(d)–(f) show the corresponding fitted factors. The static factor, Fig. S2(d), depends on both the photorecombination and the response of our spectrometer. The ionization factor, S2(e), shows the expected steep nonlinearity with intensity. The dynamical factor is shown in Fig. S2(f) for completeness, as all other plots of this quantity in this manuscript show bootstrap confidence intervals derived from a set of intensity scans.

Although the focus of this article is on dynamics, it is useful to compare the ionization factor $B(I_L)$ obtained from experimental and simulated data. This is shown in Fig. S3.

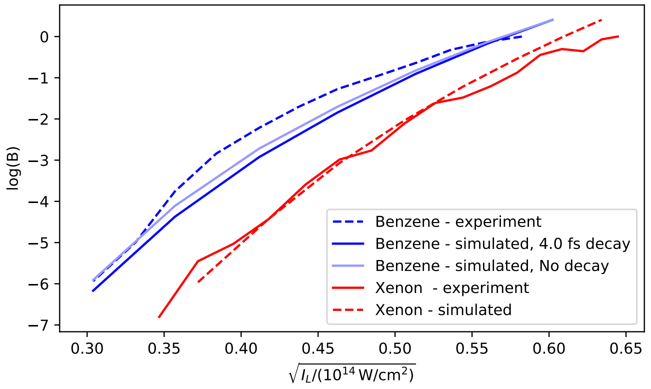

Figure S3. Ionization factor $B(I_L)$ benzene (blue) and xenon (red), experimental (dashed) and simulated (solid). For simulated benzene, curves are shown for 4 fs decay time (light blue) and no decay (dark blue).

S3 High-harmonic generation simulation

We use a form of the SFA[3] extended to high-harmonic generation in molecules by summing coherently over multiple cation states[4–8]. For completeness, our model also includes laser-induced electronic dynamics in the cation between ionization and recombination[9]. While it is in principle possible to include a full description of the vibronic dynamics of the cation in an SFA calculation[10], such calculations are at present prohibitively expensive.

In the following, boldface symbols represent spatial vectors, and a quantity with an overbar indicates it is a vector or matrix running over the cation states. For a particular molecular orientation, the high-frequency part of the dipole response at recombination time t_r is

$$\boldsymbol{D}(t_\mathrm{r}) = \int_{-\infty}^{t_\mathrm{r}} \tau^{-3/2} e^{iS(t_\mathrm{r},t_\mathrm{i})} g(\tau) \bar{\boldsymbol{d}}_\mathrm{r}^\dagger(\boldsymbol{v}(t_\mathrm{r})) \bar{U}(t_\mathrm{r},t_\mathrm{i}) \boldsymbol{E}(t_\mathrm{i}) \cdot \bar{\boldsymbol{d}}_\mathrm{i}(\boldsymbol{v}(t_\mathrm{i})) \mathrm{d}t_\mathrm{i} + \mathrm{c.c.} \tag{1}$$

The equation is an integral over ionization times t_i and the integrand, a matrix product, can be read from right to left. The factor describing the angular dependence of the ionization rate is a column vector over cation states

$$\bar{\boldsymbol{d}}_\mathrm{i}(\boldsymbol{v}) = \left(\boldsymbol{d}_\mathrm{i}^{(1)}(\boldsymbol{v}), \boldsymbol{d}_\mathrm{i}^{(2)}(\boldsymbol{v}), \ldots\right)^\mathrm{T} \tag{2}$$

where $\boldsymbol{d}_\mathrm{i}^{(n)}(\boldsymbol{v})$ is the ionization dipole of state n for momentum $\boldsymbol{v}$. The usual SFA definitions[3] apply; the kinetic momentum is $\boldsymbol{v}(t) = \boldsymbol{p} + \boldsymbol{A}(t)$ where $\boldsymbol{p}$ is the canonical stationary-phase momentum

$$\boldsymbol{p} = \frac{-\int_{t_\mathrm{i}}^{t_\mathrm{r}} \boldsymbol{A}(t)\mathrm{d}t}{\tau}, \tag{3}$$

the trajectory duration $\tau = t_\mathrm{r} - t_\mathrm{i}$, and $\boldsymbol{E}(t)$ and $\boldsymbol{A}(t)$ are the laser electric field and vector potential respectively. The τ here is the same as the Methods section of the main text, as the time window over which the cation evolves is given by the trajectory duration. The next term in the integrand of (1) is a matrix describing evolution of the cation states under the influence of the laser field. It is the solution of the Schrödinger equation

$$\frac{\mathrm{d}\bar{U}(t,t_\mathrm{i})}{\mathrm{d}t} = -i\left[\bar{I}_\mathrm{p} - \boldsymbol{E}(t)\cdot\Delta\bar{\boldsymbol{\mu}}\right]\bar{U}(t,t_\mathrm{i}) \tag{4}$$

$$\bar{U}(t_i,t_i) = \bar{I} \tag{5}$$

where the diagonal matrix $\bar{I}_\mathrm{p}$ contains the ionization potentials $I_\mathrm{p}^{(1)}, I_\mathrm{p}^{(2)}, \ldots$ on its main diagonal, $\Delta\bar{\boldsymbol{\mu}}$ is a matrix of cation state dipole couplings (the dot product $\boldsymbol{E}(t)\cdot\Delta\bar{\boldsymbol{\mu}}$ runs over the spatial coordinates) and $\bar{I}$ is the identity matrix. The recombination step is described in (1) by $\bar{\boldsymbol{d}}_\mathrm{r}(\boldsymbol{v})$, a column vector of recombination dipoles in the same format as $\bar{\boldsymbol{d}}_\mathrm{i}(\boldsymbol{v})$, and † denotes the conjugate transpose. Finally the scalar exponential contains the standard SFA action

$$S(t_\mathrm{r},t_\mathrm{i}) = -\int_{t_\mathrm{i}}^{t_\mathrm{r}} \frac{(\boldsymbol{p}+\boldsymbol{A}(t))^2}{2}\mathrm{d}t \tag{6}$$

and the $\tau^{-3/2}$ factor describes spreading of the continuum electron wavepacket.

The ionization dipole amplitude is the length-gauge plane-wave approximation

$$\boldsymbol{d}_\mathrm{i}^{(n)}(\boldsymbol{v}) = \langle\boldsymbol{v}|(1-|\psi_n\rangle\langle\psi_n|)\boldsymbol{x}|\psi_n\rangle \tag{7}$$

where $|\psi_n\rangle$ is the Dyson orbital corresponding to cation state n. The projection operator in (7) arises from our use of the dressed form of the SFA, as described by eq. (33) of Smirnova et al.[11]. This form is necessary to ensure invariance with respect to translation of the molecule. For recombination we use the plane-wave approximation

$$\boldsymbol{d}_\mathrm{r}^{(n)}(\boldsymbol{v}) = \boldsymbol{v}\langle\boldsymbol{v}|\psi_n\rangle. \tag{8}$$

We have verified that substituting spectrally-filtered photoionization cross-sections does not significantly affect the retrieved parameters (data not shown). Our calculations use the five lowest lying cation states. These are well approximated by a single hole in the neutral (the Koopmans' picture[12, 13]), and therefore throughout this work we equivalently refer to them by their corresponding molecular orbital. For the purposes of HHG, we checked this equivalence directly by comparing the spectra produced using Dyson and natural orbitals. The Dyson orbitals were calculated within the static exchange (i.e. Hartree-Fock)

model[14]. The calculation included a diffuse basis of continuum orbitals with angular momentum up to $L = 6$. The inclusion of the diffuse continuum functions has proved important for an accurate description of the exponential tail of the Dyson orbitals[15]. The natural orbitals (orbitals diagonalising the 1-electron density matrix) were calculated with the complete active space self-consistent field (CASSCF) electronic structure method state-averaging over the two equally-weighted lowest-energy cationic eigenstates. We used an active set consisting of the 6 π orbitals and 6-31G* basis set. We found insignificant differences between the harmonics calculated using the two sets of orbitals.

The factor $g(\tau)$ is the nuclear autocorrelation function which here takes Gaussian form as described in the main text.

Because the solution of equation (4) must be evaluated for every pair of ionization time t_i and observation time t, an efficient numerical implementation is essential. Because laser-induced cation state coupling plays a small role here and the laser is linearly polarized, we approximate the matrix exponential solution to (4)

$$\bar{U}(t,t_\mathrm{i}) = \exp\left\{-i(t-t_\mathrm{i})\bar{I}_\mathrm{p} - i\left[\boldsymbol{A}(t) - \boldsymbol{A}(t_\mathrm{i})\right]\cdot\Delta\bar{\boldsymbol{\mu}}\right\} \tag{9}$$

using a split-operator formula[16]

$$\bar{U}(t,t_\mathrm{i}) \approx \bar{\mathscr{I}}\exp\left\{-i\left[A(t) - A(t_\mathrm{i})\right]\hat{\boldsymbol{e}}\cdot\Delta\bar{\boldsymbol{\mu}}\right\}\bar{\mathscr{I}}. \tag{10}$$

Here, $\bar{\mathscr{I}}$ is the ionization potential operator corresponding to half the time between ionization and observation

$$\bar{\mathscr{I}} = \exp\left[-\frac{i}{2}(t-t_\mathrm{i})\bar{I}_\mathrm{p}\right], \tag{11}$$

and $\hat{e}$ is a unit vector parallel to the laser electric field. The matrix of dipole components along the electric field direction $\hat{\boldsymbol{e}}\cdot\Delta\bar{\boldsymbol{\mu}}$ is independent of t and t_i. Therefore it can be diagonalized once for each orientation of a given molecule and its exponential evaluated quickly using only scalar exponentials. A higher-order splitting would increase the accuracy but we found that equation (10) was sufficient for convergence.

The diagonal elements of the cation dipole matrix $\Delta\bar{\boldsymbol{\mu}}$ lead to dynamic Stark shifts. These elements are equal to the difference between the dipole moments of the cation states and the neutral[17–19]. The off-diagonal elements lead to laser-driven transitions between the cation states. In the Koopmans' approximation, the transition dipole $\Delta\bar{\boldsymbol{\mu}}$ is given by the single-particle matrix element:

$$\Delta\bar{\boldsymbol{\mu}}_{m,n} = \langle\psi_m|\boldsymbol{x}|\psi_n\rangle\,. \tag{12}$$

The transition dipoles from (12) are all within 0.04 au of transition dipoles calculated from the fully-relaxed cationic wavefunctions.

We have tested our model for invariance with respect to translation of the molecule[11]; to this end inclusion of dynamic Stark shifts is essential. We take the centroid of the benzene ring as the origin in all calculations.

Macroscopic trajectory selection is modelled by coherently averaging across the plane of the laser focus, which is taken as spatially and temporally Gaussian. For the temporal integration we use the adiabatic approximation[20]. The macroscopic harmonic amplitudes are taken as the amplitude of the emission on-axis ($k_\perp = 0$) and at the centre of each harmonic ($\omega = q\omega_\mathrm{l}$), which strongly favours the short trajectories. In this approximation the spectra are independent of the laser waist size and pulse duration except for a global scale factor.

References

1. Kolda, T. G. & Bader, B. W. Tensor decompositions and applications. *SIAM Rev.* **51**, 455–500 (2009).
2. De Lathauwer, L., De Moor, B. & Vandewalle, J. A multilinear singular value decomposition. *SIAM J. Matrix Anal. Appl.* **21**, 1253–1278, DOI: 10.1137/S0895479896305696 (2000). https://doi.org/10.1137/S0895479896305696.
3. Lewenstein, M., Balcou, P., Ivanov, M. Y., L'Huillier, A. & Corkum, P. B. Theory of high-harmonic generation by low-frequency laser fields. *Phys. Rev. A* **49**, 2117–2132, DOI: 10.1103/PhysRevA.49.2117 (1994).
4. Mairesse, Y. *et al.* High harmonic spectroscopy of multichannel dynamics in strong-field ionization. *Phys. Rev. Lett.* **104**, 213601, DOI: 10.1103/PhysRevLett.104.213601 (2010).
5. Smirnova, O. *et al.* Attosecond circular dichroism spectroscopy of polyatomic molecules. *Phys. Rev. Lett.* **102**, 063601, DOI: 10.1103/PhysRevLett.102.063601 (2009).
6. Smirnova, O. *et al.* High harmonic interferometry of multi-electron dynamics in molecules. *Nature* **460**, 972–977 (2009).

7. Smirnova, O., Patchkovskii, S., Mairesse, Y., Dudovich, N. & Ivanov, M. Y. Strong-field control and spectroscopy of attosecond electron-hole dynamics in molecules. *Proc. Natl. Acad. Sci. U. S. A.* **106**, 16556–16561, DOI: 10.1073/pnas.0907434106 (2009). http://www.pnas.org/content/106/39/16556.full.pdf.

8. Smirnova, O. & Ivanov, M. *Multielectron High Harmonic Generation: Simple Man on a Complex Plane*, 201–256 (Wiley-VCH Verlag, 2014).

9. Etches, A. & Madsen, L. B. Extending the strong-field approximation of high-order harmonic generation to polar molecules: gating mechanisms and extension of the harmonic cutoff. *J. Phys. B: At., Mol. Opt. Phys.* **43**, 155602 (2010).

10. Patchkovskii, S. & Schuurman, M. S. Full-dimensional treatment of short-time vibronic dynamics in a molecular high-order-harmonic-generation process in methane. *Phys. Rev. A* **96**, 053405, DOI: 10.1103/PhysRevA.96.053405 (2017).

11. Smirnova, O., Spanner, M. & Ivanov, M. Anatomy of strong field ionization ii: to dress or not to dress? *J. Mod. Opt.* **54**, 1019–1038, DOI: 10.1080/09500340701234656 (2007). http://dx.doi.org/10.1080/09500340701234656.

12. Bransden, B. H. & Joachain, C. J. *Physics of atoms and molecules* (Prentice Hall, 2003).

13. Koopmans, T. Über die Zuordnung von Wellenfunktionen und Eigenwerten zu den Einzelnen Elektronen Eines Atoms. *Physica* **1**, 104 – 113, DOI: http://dx.doi.org/10.1016/S0031-8914(34)90011-2 (1934).

14. Harvey, A. G., Brambila, D. S., Morales, F. & Smirnova, O. An r-matrix approach to electron–photon–molecule collisions: photoelectron angular distributions from aligned molecules. *J. Phys. B: At., Mol. Opt. Phys.* **46**, 215005 (2014).

15. Mašín, Z. *et al.* Electron correlations and pre-collision in the re-collision picture of high harmonic generation. *J. Phys. B: At., Mol. Opt. Phys.* **51**, 134006 (2018).

16. Bandrauk, A. D. & Shen, H. Exponential split operator methods for solving coupled time-dependent Schrödinger equations. *J. Chem. Phys.* **99**, 1185–1193, DOI: 10.1063/1.465362 (1993). https://doi.org/10.1063/1.465362.

17. Dimitrovski, D., Martiny, C. P. J. & Madsen, L. B. Strong-field ionization of polar molecules: Stark-shift-corrected strong-field approximation. *Phys. Rev. A* **82**, 053404, DOI: 10.1103/PhysRevA.82.053404 (2010).

18. Holmegaard, L. *et al.* Photoelectron angular distributions from strong-field ionization of oriented molecules. *Nat. Phys.* **6**, 428–432 (2010).

19. Li, H. *et al.* Orientation dependence of the ionization of CO and NO in an intense femtosecond two-color laser field. *Phys. Rev. A* **84**, 043429, DOI: 10.1103/PhysRevA.84.043429 (2011).

20. Varjú, K. *et al.* Reconstruction of attosecond pulse trains using an adiabatic phase expansion. *Phys. Rev. Lett.* **95**, 243901, DOI: 10.1103/PhysRevLett.95.243901 (2005).
